# Nitrogen release rates from slow- and controlled-release fertilizers influenced by placement and temperature

**DOI:** 10.1371/journal.pone.0234544

**Published:** 2020-06-17

**Authors:** Curtis J. Ransom, Von D. Jolley, Trenton A. Blair, Lloyd E. Sutton, Bryan G. Hopkins

**Affiliations:** Plant and Wildlife Sciences Department, Brigham Young University, Provo, Utah, United States of America; University of Minnesota, UNITED STATES

## Abstract

Controlled-release and slow-release fertilizers can effectively supply nitrogen (N) while mitigating N loss. To determine the suitability of these fertilizers for plants in semi-arid environments, these fertilizers need to be evaluated under varying placement and temperature conditions. Several urea fertilizers were evaluated, including: uncoated, sulfur-coated (SCU), polymer-coated-sulfur-coated (PCSCU), and polymer-coated (PCU) with projected release timings between 45 and 180 d. Nitrogen release was measured under daily fluctuating or static temperatures applied either to the surface or buried in the soil. A second experiment consisted of two PCU sources and added a hanging bag placement comparison and low and high soil moisture treatments. For the first Experiment, the N in uncoated urea released shortly after application. The SCU and PCSCU treatments released > 80% of the N before the first sampling date. With fluctuating temperatures, the PCU 45, 75, 120, and 180 incorporated into the soil released N within +9, +9, -22, and -68 d of their expected timing. However, they released their N within 35 d when surface applied. Conversely, with static temperatures, PCU products released slowly, releasing under 80% for the entire study. The second experiment verified these results and showed no difference between low and high moisture and minimal release with fertilizer not in contact with soil. Each coated fertilizer in these studies exhibited slow/control release properties, but the PCU (surface applied) and SCU/PCSCU (surface applied or incorporated in soil) release was much more rapid than expected. Our research suggests that, although the SCU and PCSCU showed minimal slow-release properties (regardless of placement), the PCU fertilizers incorporated in the soil do have a controlled release approximate to what is expected, but have a much more rapid release when surface applied.

## Introduction

Plants require nitrogen (N) to complete their life cycles. Deficient or excess N results in poor plant growth [1,2]. Plant available N can originate from soil organic matter, irrigation water, N fixation, atmospheric deposition, and fertilizer application. Applying N fertilizers in excess is a common practice to ensure plants are not N limited, especially in urban systems. Excess N not utilized by the plant contributes to a waste of natural resources and money [2–7]. Nitrogen fertilization may result in N lost to the environment through ammonia volatilization, nitrate leaching, and by-products of denitrification/nitrification. Nitrogen loss, in the various mobile forms, contributes to issues in the atmosphere and hydrosphere that ultimately affect human and animal health [2,8,9].

Research has been done on a wide variety of species to enhance N use efficiency by implementing best management practices, such as applying the right source at the right rate, placement, and timing [2,10–12]. Not following these best management practices often results in amplified N lost to the environment—degrading water and air quality [2,4,5,13].

This concern is accentuated in turfgrass systems. These systems occupy 1.9% of the surface area of the U.S. and are generally managed by homeowners who are largely unaware of potential losses resulting from mismanagement of N fertilizers and rarely see the effect of N loss as it occurs outside of their property [14]. The financial impact is not as visible to most turfgrass managers in contrast to agricultural producers. Homeowners often recognize that fertilizers result in greener lawns, but they are often uninformed on fertilizer best management practices, which may lead to over applying N fertilizers. Over application leads to N lost to the environment which degrades the atmosphere and hydrosphere. This becomes more problematic when homeowners use one of the most commonly sold fertilizers, urea [CO(NH_2_)_2_] which has a high propensity for being converted to N forms that can quickly be lost to the environment [2].

Urea consists of a carbonyl functional group with two attached amino groups.

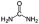


This molecular structure makes it hydrophilic with a high affinity for water—even absorbing water from humid air. When applied to the soil with even a modicum of plant-available water, it is hydrolyzed by the ubiquitous urease enzyme. Hydrolysis cleaves off the amino groups, with each picking up a proton to form NH_3_ (g), which can be volatilized into the atmosphere or, preferably, can pick up another proton to form NH_4_^+^ (aq). This process takes ~1–4 d to complete when urea is unprotected. To decrease N losses to the environment while still maintaining healthy turfgrass, the rate of urea hydrolysis needs to be limited or slowed down. This can occur by using various enhanced efficiency fertilizers (EEF) technologies [2,4]. There are a variety of EEF N fertilizers that are marketed for agronomic and horticulture crops, which are designed to release N over an extended and, ideally, a predictable period, rather than all at once. These EEFs are classified as inhibitor/stabilizer or slow/control release [2].

The inhibitor/stabilizer products delay the conversion of urea to ammonium and/or ammonium to nitrate. These delays reduce ammonia volatilization and nitrate loss from leaching or gas emission. Whereas, slow-release fertilizers (SRF) releases N based on chemical/biological action. For example, sulfur coated urea (SCU) has a coating that protects the hydrophilic urea until the sulfur is oxidized. The N in controlled-release fertilizer (CRF) is released based on physical processes. For example, the urea in polymer-coated urea (PCU) is released as pores in the coating are widened due to hydrophilic swelling and degradation of the coating—with an increasing number of molecules breaching the barrier as temperatures increase their speed of travel in the aqueous solution inside the granule. The PCU fertilizers have a more controlled rate of release based on the engineering of the coating type and thickness and are why they are classified as CRFs. Some fertilizers blend the technologies, such as with polymer-coated sulfur-coated urea (PCSCU), which is a PCU that is then coated with sulfur or vice versa. These EEFs can better match plant N needs throughout the growing season and reduce the time of exposure of N to conditions that facilitate N loss.

Nitrogen release rates from PCU is regulated by physical and abiotic constraints of the polymer type, coating thickness, soil moisture, and soil temperature [15]. By increasing the coating thickness of PCU, N release can be metered out to match plant N demand more directly [16]. However, the greatest impact on PCU’s N release comes from temperature—as the rate of diffusion doubles for about every 10°C increase [17]. In contrast, SCU relies on urea being exposed once the sulfur coating is oxidized by microbial processes and/or physically broken [15]. As a result, the release of N from SCU is variable and not as predictable as PCU [7]. With PCSCU, the outer polymer coating is less thick than with PCU, reducing the cost of using an expensive polymer while, theoretically, providing predictable N release characteristics [7]. Using these CRF and SRF have been shown to improve economic yields and quality of commodity crops significantly while reducing labor costs by reducing N application frequency, and increasing N use efficiency, and decreasing N lost to the environment [2,4,5,10,11,16,18,19].

Fertilizer recommendations using these products are partially based on the predicted N released over an amount of time and the plant’s N needs. The release timings of these products are often determined in a laboratory under static temperatures with products placed in containers of water and often stirred to maximize the driving force of diffusion [20]. A release-rate equation is calculated to estimate the longevity of these products [21]. These estimates are often adjusted to fit field conditions by using the average soil temperature or air temperature, with the release timing halved for every increase in 10°C [16]. The accuracy of these equations has been found to vary with additional research required to further understand how to better model the N release from these products under field conditions [6,17,22].

We hypothesize that these discrepancies between laboratory and field data may result from 1) fixed room temperature in contrast to widely fluctuating diurnal and seasonal shifts in temperature, 2) saturated anaerobic laboratory conditions compared to mostly unsaturated aerobic conditions in the field, 3) constant stirring resulting in friction wear on the coating and/or an increased rate of diffusion, 4) photodegradation disparities because of differences in intensity and type of light (full sun vs. partial shade under a plant canopy vs. full or partial day exposure to artificial light in a lab), and 5) equilibrium chemistry feedback mechanisms when urea concentration in the solution is unnaturally high it may affect chemical reaction and/or diffusion rates.

Of these reasons cited for discrepancies, the temperature has the largest known effect on N release from PCU fertilizers as diffusion rates highly depend on temperature when adequate moisture is available [23–25]. While it is understood that temperature is a major driving factor of N release from CRF and SRF, few studies have looked at the effect of fluctuating temperatures on N release in field conditions [26]. To match N release with plant N need, temperature fluctuations need to be considered. This is especially true for surface applications where temperatures are not buffered as much as when buried in the soil—commonly spiking to well over 40°C during direct sun exposure and dropping significantly during cold nights.

The objectives of these studies were to determine the N release rate from various SRF and CRF as a function of (a) fluctuating temperatures and (b) fertilizer placement. The initial study (experiment 1) was completed in 2011 and then largely repeated (experiment 2) in 2018.

## Materials and methods

### General experimental conditions and design

Both experiments were set up to test all factorial combinations of each fertilizer source, placement, and temperature treatments. Enough experimental units, specific to each fertilizer, were created to provide three replicates of each treatment for each sampling event. At each sampling event, three experimental units from each treatment were randomly selected and granules were destructively analyzed. The total number of experimental units created differed by fertilizer type. For example, the recommend release rate of PCU 180 was 180 d and, thus, enough experimental units were made to allow for 180 d (26 weeks) of destructive sampling; therefore, 468 experimental units were created for this fertilizer source (three replications x three positions x two temperatures x 26 weeks = 468). The same method was used to determine the number of experimental units for all other fertilizers with the number of weeks of sampling changing based on the fertilizer’s expected release timing as listed by the manufacturer. However, the testing of each treatment ceased once that treatment was shown to have released ≥ 80% of the original N amount, which is the commonly used benchmark for being fully released [16].

The soil used for both experiments was from the A_p_ horizon of a Timpanogos fine-loam, mixed, mesic Calcic Argixerolls that was gathered from the Brigham Young University (BYU) experimental farm (near Spanish Fork, UT, USA; 40°4′1.77″ N Latitude 111°37′44.99″ W Longitude; 1400 m elevation above sea level). The soil had a pH of 7.1 and 4% organic matter. The soil was air-dried and sieved through a 2-mm screen, placed in plastic 6-cm square pots to a depth of 4.5 cm with a landscape fabric liner (Weed-barrier^®^ 1 oz., DeWitt Company, Sikeston, MO, USA) to prevent soil loss from drainage holes.

#### Experiment 1: Treatments

Fertilizer source treatments included: 1) uncoated urea (46-0-0), 2) SCU (39-0-0-19S), 3) XCU^©^ made up of urea coated with polymer and an outer coating of elemental sulfur and wax (PCSCU; 41-0-0-4S), 4) Duration CR^©^ 45 (PCU 45; 43-0-0), 5) Duration CR^©^ 75 (PCU 75; 43-0-0), 6) Duration CR^©^ 120 (PCU 120; 43-0-0), and 7) Duration CR^©^ 180 (PCU 180; 43-0-0). The PCSCU and PCU products were supplied by Nutrien [formerly (Agrium Advanced Technologies; Loveland, CO, USA); currently these products are from Koch Fertilizer, LLC (Wichita, KS, USA)] while the SCU was obtained from a bulk supply provided by the BYU Grounds Department (no manufacturer information was available). The PCU products have estimated release timings as indicated by the number in the name (i.e. PCU 45 has an estimated release timing of 45 d, PCU 75 of 75 d, etc.) and the PCSCU has an estimated release time of 45 d. The release timings are based on the time required to release 80% or more of the N in the fertilizer based on laboratory trials at 20°C in constantly stirred water baths [16]. The coated fertilizers used in this study are not necessarily representative of all other products commercially available due to differences in manufacturing procedures and materials used.

Placement treatments of the fertilizer sources included: 1) soil incorporation at a depth of 2.5 cm, 2) placement on the bare soil surface, and 3) placement on the surface of a Kentucky bluegrass thatch layer (approximately 1.5 cm thick). For thatch treatments, 4.5 cm depth of Kentucky bluegrass sod, grown on the same soil, was cut to fit the size of each pot and the canopy was cut to a height of 2.5 cm. The grass was mostly killed by desiccation to avoid the confounding effects of plant N uptake and yet still having some shading from grass shoots. The thatch treatments provided a layer between the fertilizers and the soil, which minimized moisture supplied by the soil to the granules. For treatments where fertilizer was incorporated, the soil was added to pots to a height of 2 cm and granules were placed on the soil and then covered with 2.5 cm of soil. For all treatments, granules were placed in rows spaced about 0.5 to 1 cm away from the wall of the container and adjacent granules to avoid unrealistically high fertilizer concentrations that may impact the physical chemistry of diffusion. For each experimental unit, 16 granules were counted and weighed (average mass = 0.29 g with a range of 0.21 to 0.39 g) before placing them in or on the soil.

**Fig 1:**
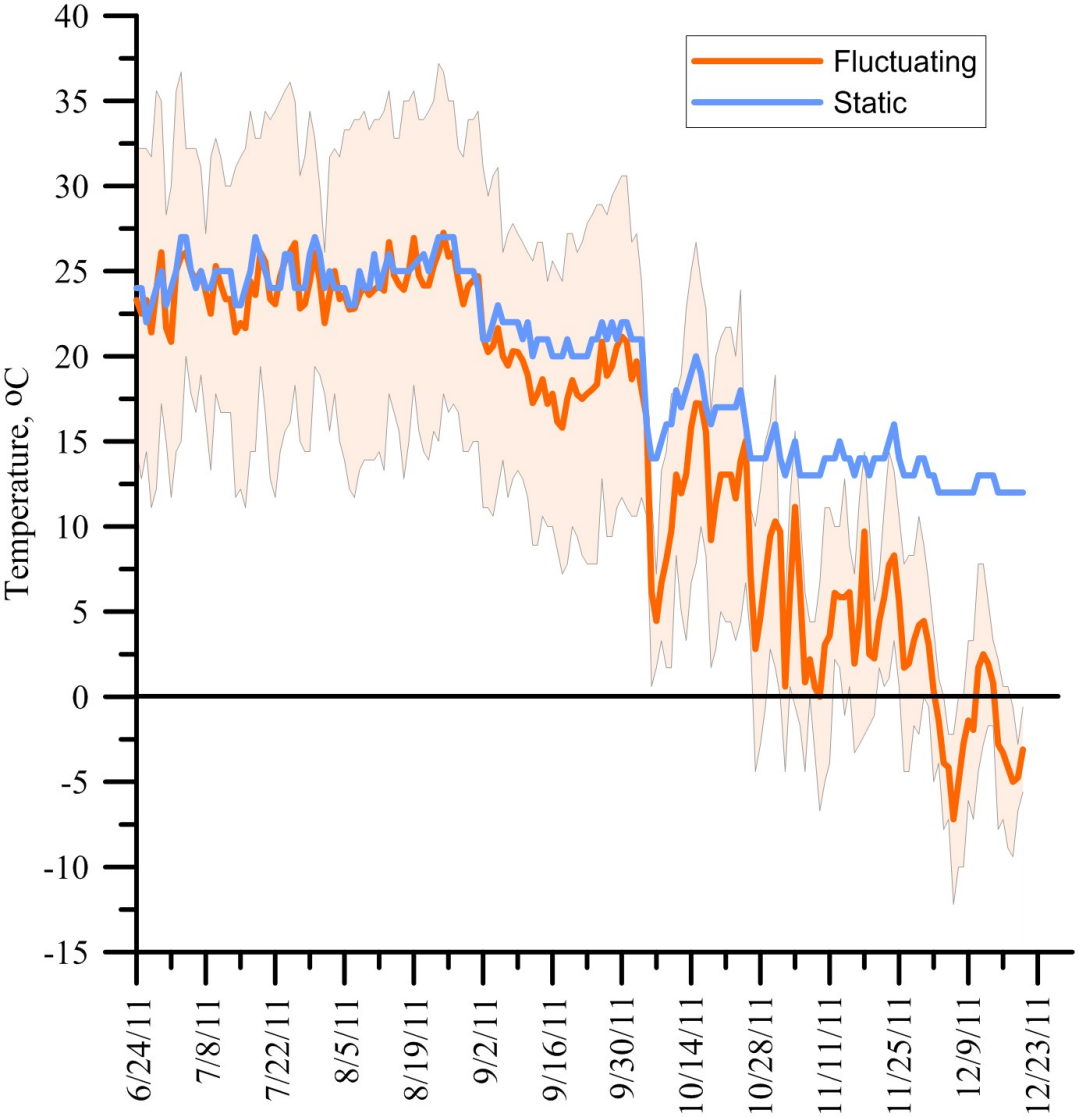
Experiment 1 air temperatures recorded from 24 June 2011 to 22 December 2011. The solid orange line indicates the average daily outdoor (fluctuating) temperatures with the borders of the shaded region indicates the maximum and minimum daily temperatures. The solid blue indicates the average daily indoor (static) temperatures.

Temperature treatments included: 1) fluctuating diurnal temperatures under field conditions with direct sunlight and 2) minimal diurnal fluctuating temperatures. The pots under the fluctuating temperature regime were placed at the BYU experimental farm where the soil was gathered from. The pots receiving the static temperature regime were placed in a storage facility without insulation or air conditioning/heating on the BYU campus (Provo, UT, USA; 40°15'51.29"N Latitude 111°39'32.11"W Longitude; 1387 m elevation above sea level). The field condition temperatures were measured using a nearby weather station (~ 60 m away from the study area; Utah State University Climate Center) that contained a thermistor placed 2.5 cm below the soil surface and logged every 8 h using an AM400 data logger (MK Hansen, Wenatchee, WA, USA). Field temperatures at the start of the study averaged at 23.5°C and ended at -3.6°C (Fig 1). The temperatures fluctuated on average +/-8.9°C around the average daily temperature. Although not directly measured in the plot area for this study, the temperature extremes at the soil surface would be expected (based on experiment 2) to be roughly double the average air temperatures during the summer months of this trial, reaching highs above 50°C. While soil temperature at the depth of the incorporated granules was not measured during this study due to faulty wires; subsequent measurements in prior years with thermistors placed at 10 cm showed a smaller magnitude of fluctuation. This is consistent with principles of thermal diffusivity and soil heat capacity resulting in a dampening of temperature fluctuations with increasing depth [27]. The average daily air temperatures for both treatments were mostly similar throughout the study, with the main difference being the daily highs and lows. The indoor temperatures were measured using a similar thermistor and logger. The first 75 d (24 June-7 September) maintained a similar average daily temperature of +/-1°C between the indoor and field condition temperatures. However, after 7 September the temperature difference between the two locations deviated > 2°C consistently (Fig 1). Indoor temperatures stayed warmer and never dropped below 10°C for the length of the project.

The study was conducted beginning June 24, 2011, and ending December 22, 2011—simulating a common scenario when CRFs are applied early summer and extending to a date when the longest expected release fertilizer product (180 d) should have completed its target N release. The soil and fertilizer in each pot were watered every 7 d (~20–25 ml/pot) by misting each pot with a hand sprayer for indoor treatments and with an overhead sprinkler system for outdoor treatments. The outdoor treatments were also subject to dew and precipitation events, but both temperature regimes were characterized by the soil having adequate moisture above permanent wilting point throughout the study.

#### Experiment 1: N release measurements

At the time of sampling, all applied granules from each pot to be measured were carefully hand removed and briefly rinsed to remove soil particles and placed into desiccators at 20–22°C for 72 h. We chose to not use the method of weighing granules [21,22,28,29] because of moisture and volatilization concerns. Instead, granules were inserted into a dilute HCl (pH 5.6) solution made with double deionized water in a 13 ml plastic vial, filled completely with essentially no atmospheric headspace and stored at 5–10°C for up to 120 d. At the time of analysis, solutions were removed from cold storage, diluted to a volume of 145 ml with a 0.28 N sodium acetate buffer at pH 4.7 to maintain an acidic pH to limit NH_3_ volatilization potential. Fertilizer granules were split open using tweezers to insure complete diffusion. Urease powder (~0.05 g) was added to catalyze urea conversion and solutions were stored for 7 d at 20–22°C to facilitate the complete conversion of urea to NH_4_^+^. Kissel [30] showed a complete conversion at 4 d at 27°C, and we observed complete conversion at 7 d at 22°C.

Before analysis of the solutions, each vial was shaken by hand for 60 s and left on a flat surface for a minimum of 60 s to allow any undissolved urease to settle out of solution. One ml was removed from the middle of the vial and diluted with 49 ml of dilute HCl (pH 3.0). Analysis of NH_4_^+^ and NO_3_^-^ was done colorimetrically with a Flow Injection Analyzer (Lachat Instruments QuikChem 8500 Series 2, Loveland, CO, USA). Process blanks were used to validate minimal N contamination throughout the procedure. As expected, due to conditions not being conducive for nitrification, NO_3_^-^N was negligible in the solutions and, therefore, only NH_4_-N was used to estimate N release rate. The amount of N released from each batch of granules was determined by subtracting the concentration found in solution from the initial weight of the fertilizers.

Before the completion of each NH_4_^+^ analysis, pH measurements were made for each stored sample, to determine the effectiveness of the buffer solution to maintain pH < 7. To conserve resources, earlier sampling date analyzes were used to determine how much buffer to add for proceeding sampling dates. Any fertilizer type that resulted in a final pH above 7 received a greater concentration of buffer (0.31 N), while those fertilizer types that had a minimal change in pH, received a lower buffer concentration (0.14 N). Any fertilizer types that were shown to have a change in pH, but did not pass pH 7, received no change in buffer concentration (0.28 N) for the following sampling date. This was continually monitored, and each analysis was adjusted accordingly.

#### Experiment 2: Treatments

A second experiment was undertaken to validate the findings from the first experiment. The soil and methods were similar. Any modifications are described below.

Fertilizer source treatments included two of those used in Experiment 1, namely: PCU 75 and PCU 120. Placement treatments of the fertilizer sources included: 1) soil incorporation at a depth of 2.5 cm, 2) placement on the bare soil surface, and 3) in a hanging bag. The hanging bag consisted of 8 cm x 10 cm nylon mesh bag with square (0.5 mm) holes. These were small enough to limit granules from falling out but large enough to provide adequate air and moisture movement. The placement of fertilizer in the soil or on the surface was similar to Experiment 1. For each experimental unit, 16 granules were counted and weighed (average mass = 0.23 with a range of 0.17 to 0.30) before placing them in or on the soil or in a hanging bag.

**Fig 2:**
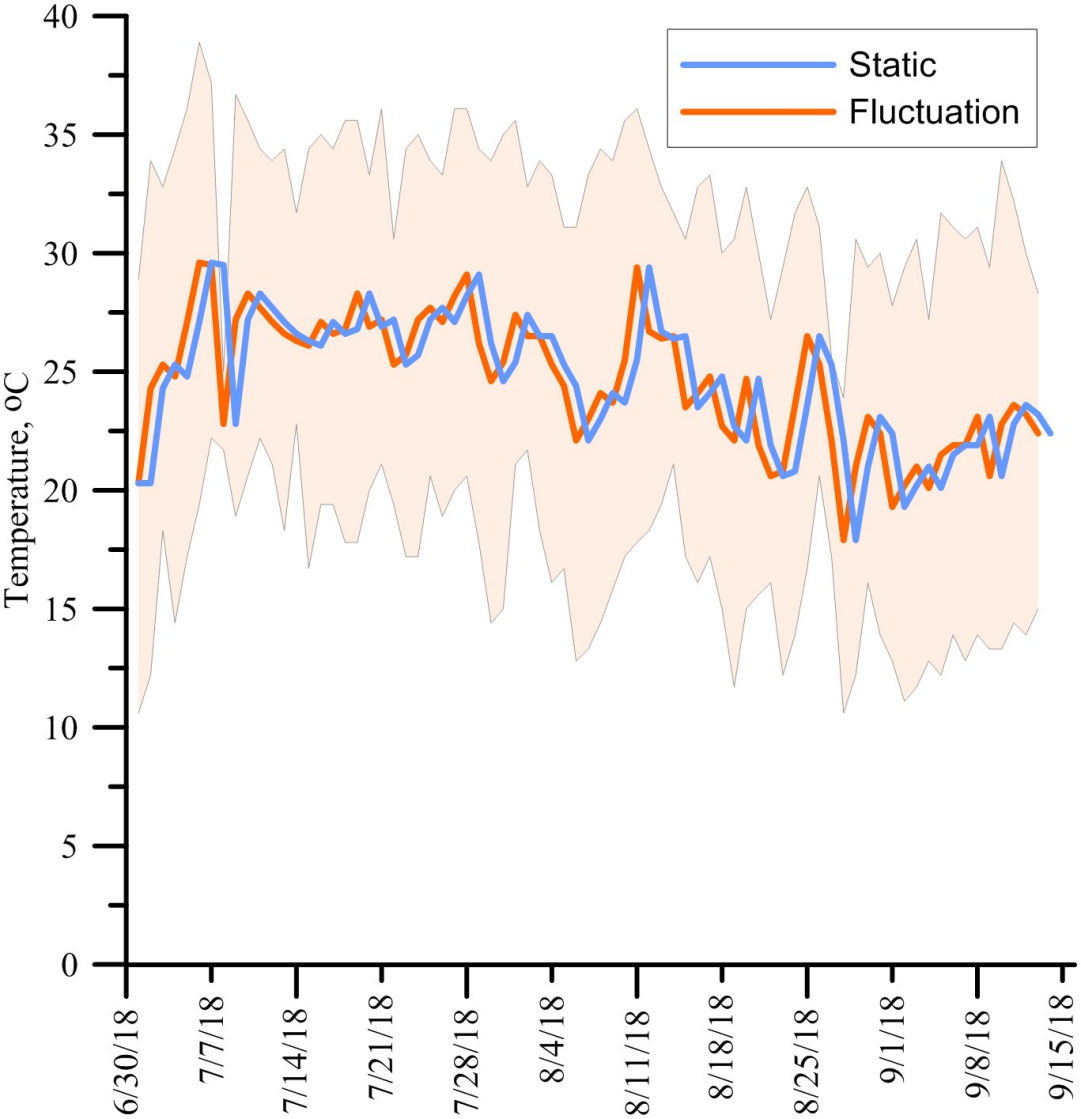
Experiment 2 air temperatures recorded from 30 June 2018 to 14 September 2018. The solid orange line indicates the average daily outdoor (fluctuating) temperatures with the borders of the shaded region indicates the maximum and minimum daily temperatures. The solid blue indicates the daily indoor (static) temperatures, which were manually set to match the average outdoor temperature from the previous day. Samples were taken 24 (24 and 25 July), 45 (15 and 16 August), and 75 (13 and 14 September) days after application.

Temperature treatments were similar to Experiment 1 with fluctuating outdoor or static indoor regimes. However, in this experiment, the experimental units with the indoor static temperature treatment were placed in an environmentally controlled growth chamber (BYU Life Sciences Building, Provo, UT, USA; 40°15′51.29″ N Latitude 111°39′32.11″ W Longitude; 1400 m elevation above sea level) to precisely match the average outdoor temperature. In this way, the only substantive difference was the magnitude of the daily high and low temperatures and not average temperature. The experimental units with outdoor fluctuating temperature treatments were placed near the BYU Life Sciences Greenhouse Complex (Provo, UT, USA; 40°15′51.29″ N Latitude 111°39′32.11″ W Longitude; 1400 m elevation above sea level). These treatments were started a day before the indoor treatments so the growth chamber’s temperature could be changed daily to match the outside average temperature from the previous day (Fig 2). The temperatures were measured using a thermistor with a data logger (Meter Group, Pullman, WA, USA). Outdoor temperatures were measured at a nearby weather station (~ 90 m away from the study area). Field condition temperatures initially started with a daily average daily of 23.1°C and ended at 23.3°C (Fig 2). The temperatures fluctuated on average +/-7.3°C around the average daily temperature. The outdoor soil temperatures at 2.5 cm below the surface fluctuated, although minimally. These started at an average of 18.3°C, peaked at 19.3°C and fell back to 18.5°C by the end of the study with an average daily fluctuation of +/-2.3°C. The outdoor soil temperatures at the surface fluctuated widely. These started at an average of 18.8°C, peaked at 20.3°C and fell back to 18.7°C by the end of the study with an average daily fluctuation of +/-17.4°C. The lows at night mostly mirrored the air temperatures, but the highs during the days were frequently greater than 50°C during days with full sun, with an average of 44.7°C. The indoor soil temperatures did not fluctuate at or below the surface, following the air temperature closely at +/-1°C.

Two moisture treatments were introduced in this experiment, which included: 1) low moisture (watered once per week—similar to Experiment 1) or 2) high moisture (watered three times per week). The soil and fertilizer were watered in the same manner done in Experiment 1 for the indoor treatments (overhead irrigation was not used in this study). As in Experiment 1, the outdoor treatments were subject to dew and precipitation events but, again, the soil had adequate moisture above the permanent wilting point throughout the study for all experimental units.

The study was initiated on 30 June or 1 July 2018 and the last sampling event occurred on 13 or 14 September 2018 (75 d after application) for the outdoor (fluctuating) and indoor (static) treatments, respectively.

#### Experiment 2: N release measurements

Rather than the weekly sampling in Experiment 1, the granules were sampled at 24, 46, and 75 days after the start of the experiment. At the time of sampling, all granules from each pot were carefully hand removed and briefly rinsed to remove soil particles. Granules were then placed in a 50 ml glass vial and were split open using tweezers. Each vial was filled using ultra-pure deionized water. Samples were then analyzed for total N using an Elemental Analyzer [Vario EL Cube (CN), Elementar, Langenselbold, Germany]. This method was validated as a quicker and less expensive method for analyzing N release compared with what was done in the first experiment. Therefore, we feel confident in being able to compare results from both experiments even with the change in methods.

### Statistical analysis

Data outliers, especially from Experiment 1, were identified for each treatment combination and removed based on Cooks’ distance values (> 4/n) and standardized residual values. Testing for treatment differences was done using an analysis of variance (ANOVA) by modeling N release as a function of fertilizer, position, temperature, and time with all four-way, three-way, and two-way interactions. Based on significant interactions, differences were calculated between treatments within a fertilizer type and determined using a pairwise comparison based on the least-squares estimates with the ‘emmeans’ package in R [31]. The Tukey’s Honest Significant Difference test was used to delineate significant differences between the slope coefficients (α = 0.05). All analyses were done using R (R Project for Statistical Computing, http://www.r-project.org/).

## Results

After removing outliers (n = 40 out of 1079 data points), the ANOVA model showed all four-way, three-way, two-way interactions, and main effects (except position) were significant (*P* ≤ 0.05). Each treatment (i.e., fertilizer source, placement position, temperature regime, and moisture level) was analyzed separately using linear regressions to model N release as a function of time in. Minimal differences in N release between all fertilizer types for granules placed on bare soil and thatch were observed. These treatments were combined after verifying a lack of distinction using a non-orthogonal contrast. All statistical analyzes were rerun using this simplified model combining bare soil and thatch placement into a “surface applied” treatment compared to the “incorporated” treatment. Similarly, from the second experiment, there was no effect (P < 0.001) of the two moisture treatments on N release regardless of fertilizer type (i.e., PCU 75 or PCU 120), placement (i.e., hanging bag, surface applied, or incorporated), or temperature (i.e., static or fluctuating). All analyzes in experiment 2 were done using all the observations from the moisture treatments combined.

**Fig 3:**
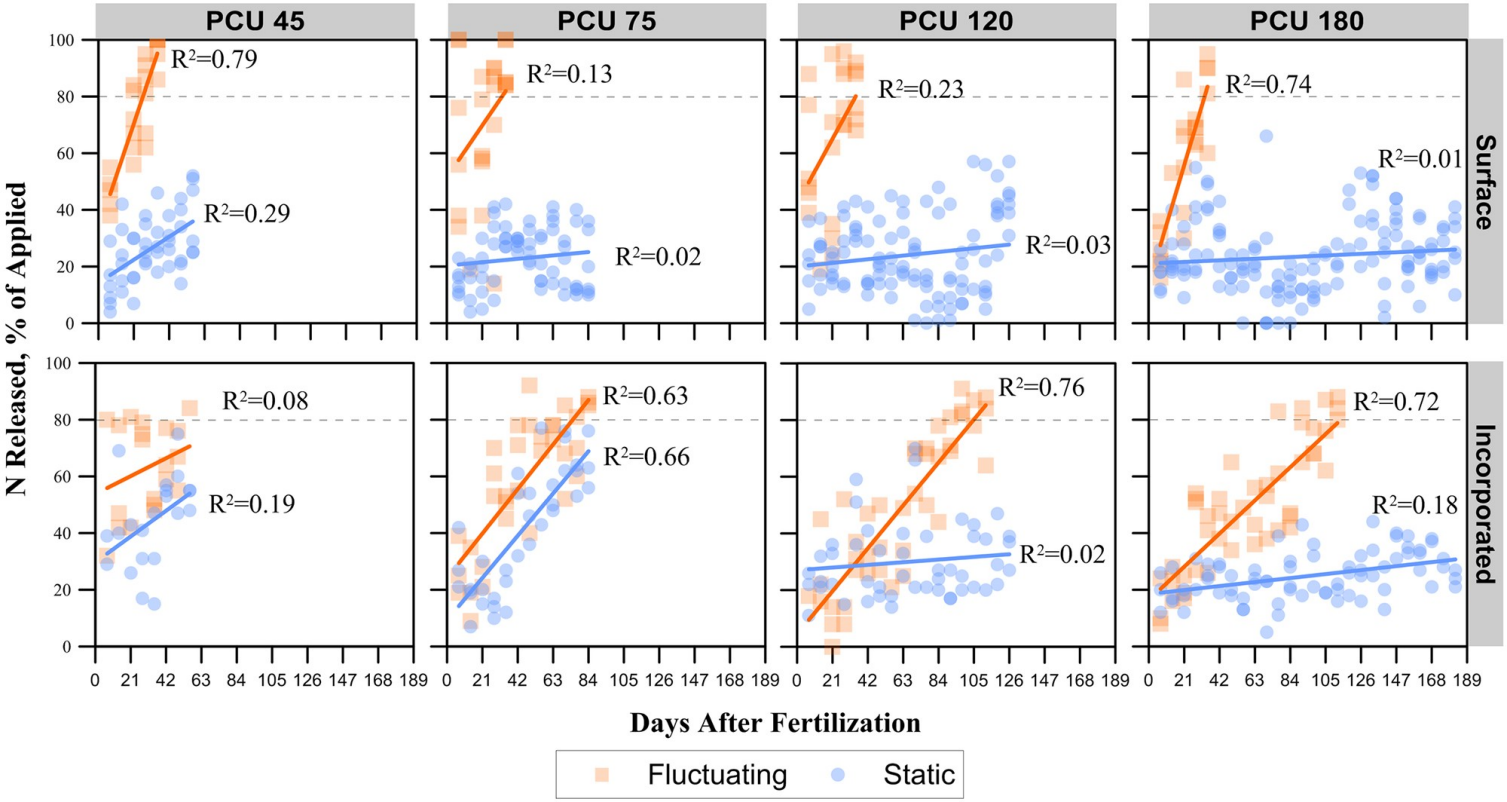
Experiment 1 N release rates for various fertilizer sources. (PCU) polymer-coated urea products with varying targeted release timings of 45, 75, 120, and 180 days applied either to the soil/thatch layer surface or incorporated into the soil at either static or fluctuating daily temperatures. The dashed vertical line indicates the targeted cumulative release percentage.

**Fig 4:**
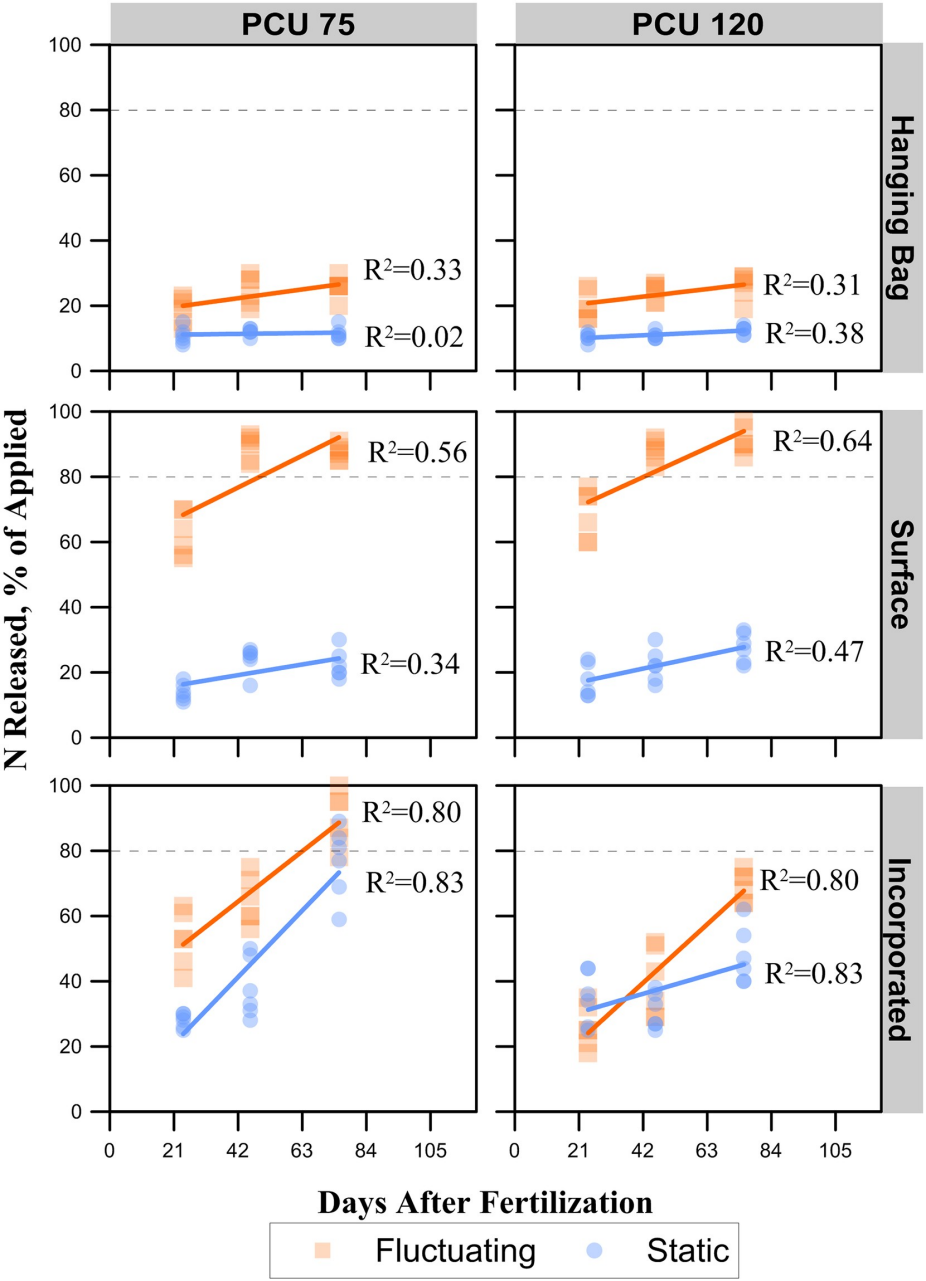
Experiment 2 N release rates for two fertilizer sources. (PCU) polymer-coated urea products with targeted release timings of 75 and 120 days applied either to the soil layer surface, incorporated into the soil, or in a hanging bag at either static or fluctuating daily temperatures. The dashed vertical line indicates the targeted cumulative release percentage.

**Fig 5:**
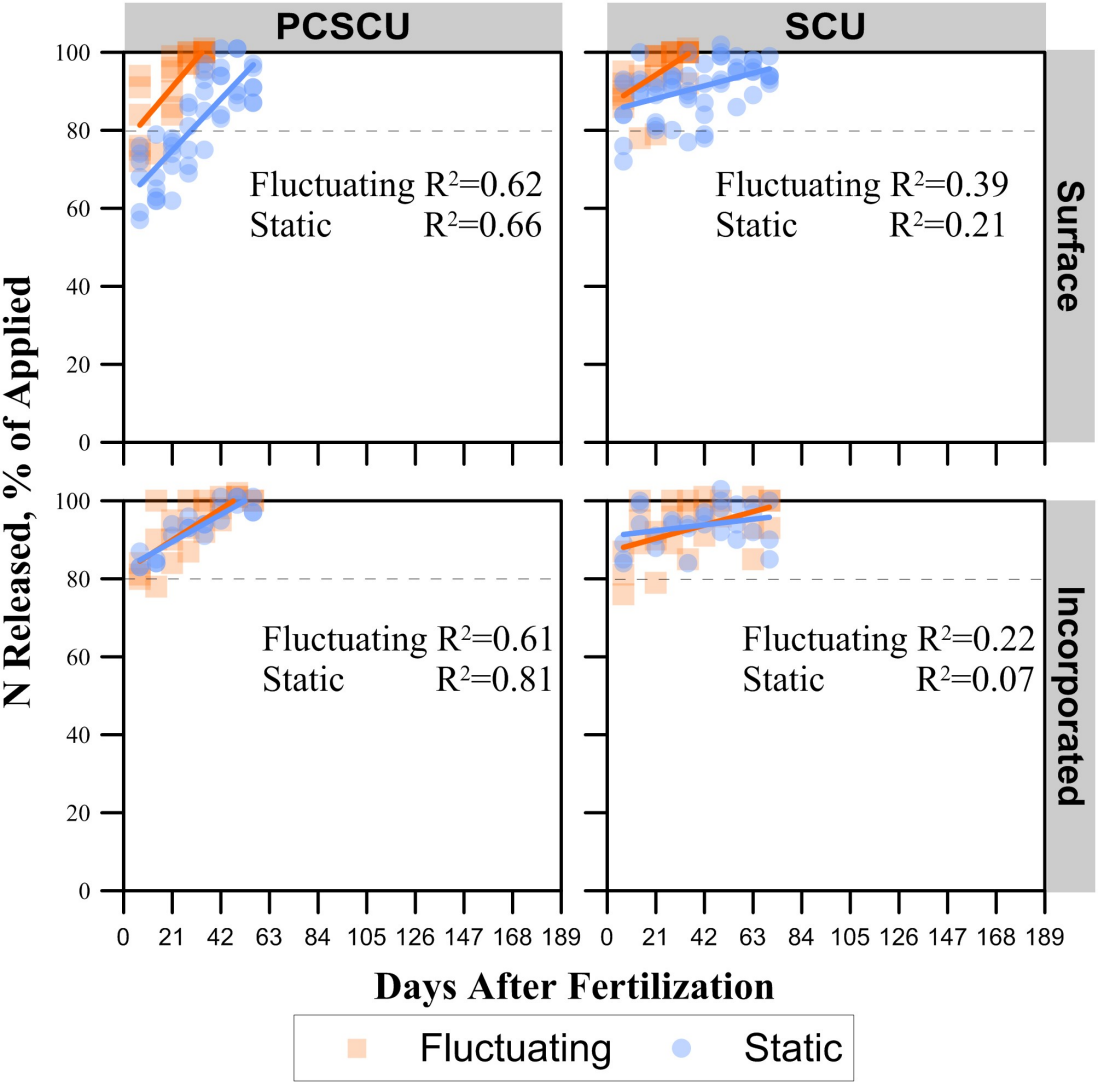
Experiment 1 N release rates for various fertilizer sources. (PCSCU) polymer-coated sulfur-coated urea, and (SCU) sulfur coated urea applied either to the soil/thatch layer surface or incorporated into the soil at either static or fluctuating daily temperatures. The dashed vertical line indicates the targeted cumulative release percentage.

Experiment 1 results are shown in Figs 3 and 5 with the corresponding comparison of slopes in Table 1. Experiment 2 results are shown in Fig 4 with the corresponding comparison of slopes in Table 2.

**Table 1:** Experiment 1 comparison of slopes of linear regression models used to model N release as a function of time for each fertilizer type and treatment combination for a study with various fertilizer sources.

			Group	**Surface**		**Incorporated**	
				Fluctuating	Static	Fluctuating	Static
**PCU 45**	**Surface**	Fluctuating	A	**y = 33 + 1.77x**			
		Static	D	**	**y = 14 + 0.39x**		
	**Incorporated**	Fluctuating	B	**	**	**y = 54 + 0.30x**	
		Static	C	**	**	**	**y = 30 + 0.43x**
**PCU 75**	**Surface**	Fluctuating	A	**y = 51 + 0.87x**			
		Static	D	**	**y = 20 + 0.06x**		
	**Incorporated**	Fluctuating	B	**	**	**y = 24 + 0.75x**	
		Static	C	**	**	**	**y = 9 + 0.71x**
**PCU 120**	**Surface**	Fluctuating	A	**y = 42 + 1.09x**			
		Static	C	**	**y = 20 + 0.06x**		
	**Incorporated**	Fluctuating	B	**	**	**y = 4 + 0.72x**	
		Static	C	**	NS	**	**y = 27 + 0.04x**
**PCU 180**	**Surface**	Fluctuating	**A**	**y = 14 + 2.00x**			
		Static	C	**	**y = 21 + 0.03x**		
	**Incorporated**	Fluctuating	B	**	**	**y = 16 + 0.56x**	
		Static	C	**	NS	**	**y = 19 + 0.07x**
**PCSCU**	**Surface**	Fluctuating	**A**	**y = 76 + 0.70x**			
		Static	B	**	**y = 62 + 0.63x**		
	**Incorporated**	Fluctuating	A	NS	**	**y = 82 + 0.38x**	
		Static	A	NS	**	NS	**y = 82 + 0.33x**
**SCU**	**Surface**	Fluctuating	A	**y = 86 + 0.38x**			
		Static	B	**	**y = 85 + 0.16x**		
	**Incorporated**	Fluctuating	B	*	NS	**y = 87 + 0.16x**	
		Static	B	*	NS	NS	**y = 91 + 0.07x**

(PCU) polymer-coated urea products with varying targeted release timings of 45, 75, 120, and 180 days, (PCSCU) polymer-coated sulfur-coated urea, and (SCU) sulfur coated urea applied either to the soil/thatch layer surface or incorporated into the soil at either static or fluctuating daily temperatures. Comparisons are only made within a fertilizer type. Difference between slopes was identified using NS, *, or ** to represent not significant, significant at P-values < 0.05, significant at P-values < 0.0001, respectively. This was summarized for each fertilizer source in the “Group” column with treatments not sharing any letters (A-D) differing significantly from each other.

**Table 2:** Experiment 2 comparison of slopes of linear regression models used to model N release as a function of time for each fertilizer type and treatment combination.

			Group	**Hanging Bag**		**Surface**		**Incorporated**	
				Fluctuating	Static	Fluctuating	Static	Fluctuating	Static
**PCU 75**	**Hanging bag**	Fluctuating	D	**y = 17 + 0.13x**					
		Static	E	**	**y = 11 + 0.01x**				
	**Surface**	Fluctuating	A	**	**	**y = 57 + 0.46x**			
		Static	D	NS	*	**	**y = 13 + 0.16x**		
	**Incorporated**	Fluctuating	B	**	**	*	**	**y = 34 + 0.73x**	
		Static	C	**	**	**	**	**	y **= 1 + 0.97x**
**PCU 120**	**Hanging bag**	Fluctuating	D	**y = 18 + 0.11x**					
		Static	E	**	**y = 9 + 0.04x**				
	**Surface**	Fluctuating	A	**	**	**y = 62 + 0.43x**			
		Static	D	NS	*	**	**y = 13 + 0.20x**		
	**Incorporated**	Fluctuating	B	**	**	**	**	**y = 4 + 0.86x**	
		Static	C	**	**	**	**	*	**y = 25 + 0.27x**

(PCU) polymer-coated urea products with targeted release timings of 75 and 120 days] applied either to the soil layer surface, incorporated into the soil, or in a hanging bag at either static or fluctuating daily temperatures. Comparisons are only made within a fertilizer type. Difference between release rates were identified using NS, *, or ** to represent not significant, significant at P-values < 0.05, significant at P-values < 0.0001, respectively. This was summarized for each fertilizer source in the “Group” column with treatments not sharing any letters (A-E) differing significantly from each other.

### PCU incorporated in field conditions with fluctuating temperatures

Polymer-coated urea incorporated into the soil under field conditions and exposed to fluctuating diurnal temperatures showed an effectively controlled release pattern (Fig 3), similar to the expected release pattern with PCU 45, 75, and 120 releasing > 80% of applied N after 56, 84, and 98 d, respectively. These rates were within 9 to 22 d of the projected timing of the release, with PCU 45 and 75 taken longer than expected and PCU 120 taking less time. However, PCU 180 released > 80% of N by 112 d, which is 68 d ahead of predicted. For experiment 2, PCU 75 released N > 80% between the first and second sampling dates (between 24 and 46 d after application). Whereas PCU 120 did not release > 80% within this experiment’s sampling time frame (75 d). Using the slope of the fit line, it is expected that PCU 120 would release > 80% by 93 d (Fig 4 and Table 2), similar to what was seen with experiment 1 (Fig 3). Compared to other treatments, the rate of release of PCU when incorporated with fluctuating temperatures was significantly greater than the same fertilizers incorporated or surface applied under static temperature conditions. However, the rate of release was also significantly less than the same fertilizers that were surface applied with fluctuating temperatures (Tables 1 and 2).

### PCU surface applied in field conditions with fluctuating temperatures

Polymer-coated urea applied to surface under field conditions with fluctuating diurnal temperatures resulted in the fastest release rates of all PCU treatments. For experiment 1, all PCU fertilizers released > 80% N within the first 35 d after application regardless of the targeted time of release (Fig 3). Similarly, for experiment 2, both PCU 75 and 120 released > 80% N between the first and second sampling dates (between 24 and 46 d after application), with a large majority released after 24 d (Fig 4). The release rates for all PCU products applied to the surface with fluctuating temperatures were significantly greater than all other treatments (Tables 1 and 2).

### PCU incorporated at static temperatures

Incorporated PCU granules in the soil under static temperatures did not release > 80% N. However, the PCU fertilizers with the shortest targeted release rates (i.e., PCU 45 and 75) were close to releasing > 80% N during the period N release was monitored (Fig 3). In contrast, the longer release rates (i.e., PCU 120 and 180) showed a minimal increase in N release during the period they were monitored (Fig 3). In experiment 2, a similar response was seen for PCU 75, where it came close to releasing > 80% 76 d after fertilizing. For PCU 120 in experiment 2, it was similar to experiment 1 in that it would not have reached > 80% N released within the target time frame (based on the slope coefficients; Table 2), even if we had continued sampling. In contrast to experiment 1, the rate of release was greater in experiment 2 (slope of 0.27 vs 0.04; Table 2). The release rates for PCU products incorporated at static temperatures were less than all fertilizers under fluctuating temperatures (Tables 1 and 2). These PCU treatments were had N release rates greater or equal (experiment 1 PCU 120 and 180) to fertilizers that were surface applied under static temperatures (Tables 1 and 2).

### PCU surface applied at static temperatures

Surface applied PCU fertilizers stored indoors under static temperatures did not release N > 80% for the duration of experiment 1 (Fig 3). The PCU 45 had a slope term of 0.39, whereas the other fertilizers had slope terms < 0.06 (Table 1). With additional sampling dates, it is expected that the PCU 45 would eventually release > 80% N within a season, but the others would require more time before they eventually released the majority of their N. For experiment 2, the PCU 75 and 120 had increased slope terms over experiment 1 but still did not release > 80% within the sampling period (Fig 4). This treatment tended to have the lowest release rate of all treatments, with exception to the hanging bag treatment under static temperature (Tables 1 and 2).

### Urea, SCU, and PCSCU

Urea, as expected, released 100% of its N by the first sampling date as there were no granules left to collect. The SCU exhibited minimal slow-release properties, as > 80% N was released by the first sampling date (Fig 5). Although PCSCU treatments exhibited somewhat better slow/controlled release properties compared to urea and SCU (Fig 5), it was minimal compared to PCU (Fig 3). When comparing across position and temperature treatments, the N release rate of surface-applied fertilizers under static conditions was significantly less than all other treatments. While N release rates for all other treatments were statistically similar (Table 1).

### Hanging bag

The PCU products placed in hanging bags showed minimal release characteristics (Fig 4). Across both fertilizer types, no fertilizer released > 30% N, and the slopes did not exceed 0.13. Fertilizers in hanging bags under fluctuating conditions had an increased rate of release over fertilizers in hanging bags under static conditions (Table 2). However, the hanging bag treatment (regardless of temperature regime) had the lowest release rate of all treatments (Table 2).

## Discussion

### Effects of temperature, placement, and moisture on PCU

The mode of action for PCU fertilizers requires that water diffuse into the fertilizer granule and then the dissolved urea to diffuse out of the granule into the soil. The rate of water and urea diffusion depends on temperature, with the rate doubling with every 10°C change [17]. Typically, the average air temperature is used to predict release rates of PCU fertilizers [19,24,32]. However, the results from this study show inconsistencies with using the average air temperature in modeling N release. Average air temperatures for both fluctuating and static temperature treatments were within 1 to 2°C of each other for the first 75 d (Figs 1 and 2). But our data indicate that the fluctuating temperatures of field conditions and/or high maximum daily temperatures influenced the N release rate more than the average air temperature. This occurred whether the granules were incorporated into the soil or, especially, applied to the soil or thatch surface.

Besides temperature changes, moisture levels affect N release rates from CRF, as moisture is essential to the dissolution and outward movement of N from CRF. Research indicates that a level of 50% field capacity or less will slow the release of N [33–35], yet others’ results show that high soil humidity is often enough to ensure diffusion—even when soil moisture is below 50% field capacity [23,25,36]. However, unpublished data from Nutrien (formerly Agrium at the time of the initial study) indicates that release rates were not limited until about 25 to 30% of field capacity (Alan Blaylock, personal communication). Regardless, moisture can be a limiting factor at some level.

Moisture did not seem to be a limiting factor in these studies where the granule was in direct contact with the soil or thatch. The release rates were relatively higher for treatments in the field with fluctuating temperatures. It could be argued that these had more total moisture at times due to dew condensation most mornings, and very few instances of natural precipitation. Although these were subject to greater drying due to wind and extreme temperatures during the day. Regardless, the release rates of the indoor treatments with static temperatures had lower N release compared to fluctuating temperatures, regardless of placement. And slopes for these treatments were mostly similar (Tables 1 and 2). Although the surface soil would dry out in between waterings, the weekly watering was adequate to sustain N release as we saw a minimal difference between moisture treatments. The granules incorporated were surrounded by soil with at least some available moisture above 50% of field capacity for the entire study (based on measure field capacity and ET rates). Despite adequate moisture, the N release from the static temperature with incorporated granules was very low. This strongly suggests that temperature fluctuation was the driving force for N release in these studies.

### Effects of temperature, placement, and moisture on SCU and PCSCU

In contrast to CRF, which depends on temperature and soil moisture content, SRF further depends on microbial activity and/or chemical processes to breakdown coatings [37]. While a slow-release of N was expected from SCU, this study showed minimal slow-release properties. Although over 80% of the N was released from the SCU, regardless of placement method or temperature fluctuation, by the first sampling date, there was a continued release pattern with an identifiable slope (Fig 4 and Table 1). This suggests that the N released more slowly than uncoated urea that disappeared visually within hours after being applied. All SCU granules were observed to be physically cracked after 7 d after application. Not all SCU fertilizers would be expected to behave similarly as studies have reported release timings between 3 and 126 d [38–40]. The SCU fertilizer used in this study did not perform much differently than uncoated urea. However, even a few days delay may substantively reduce ammonia volatilization [41].

The combination of both SCU and PCU coatings in a PCSCU fertilizer did not have as long of an extended-release as was expected, although there appeared to be some controlled/slow-release properties. This is contrary to what was expected with the manufacturer stating a 45 d release. The sulfur coating may have worked similar to the SCU, but it is curious as to why the polymer coating did not perform at least somewhat similar to the PCU products. These polymer coatings for PCSCU fertilizers are very thin. This reduces the total cost over a PCU product. It is possible that this very thin coating was harmed by the sulfur coating or the thin coating provided only minimal control release timing. Regardless, as with SCU, there was a significant impact on PCSCU with temperature, placement, and moisture.

### Implications for N management

**Fig 6:**
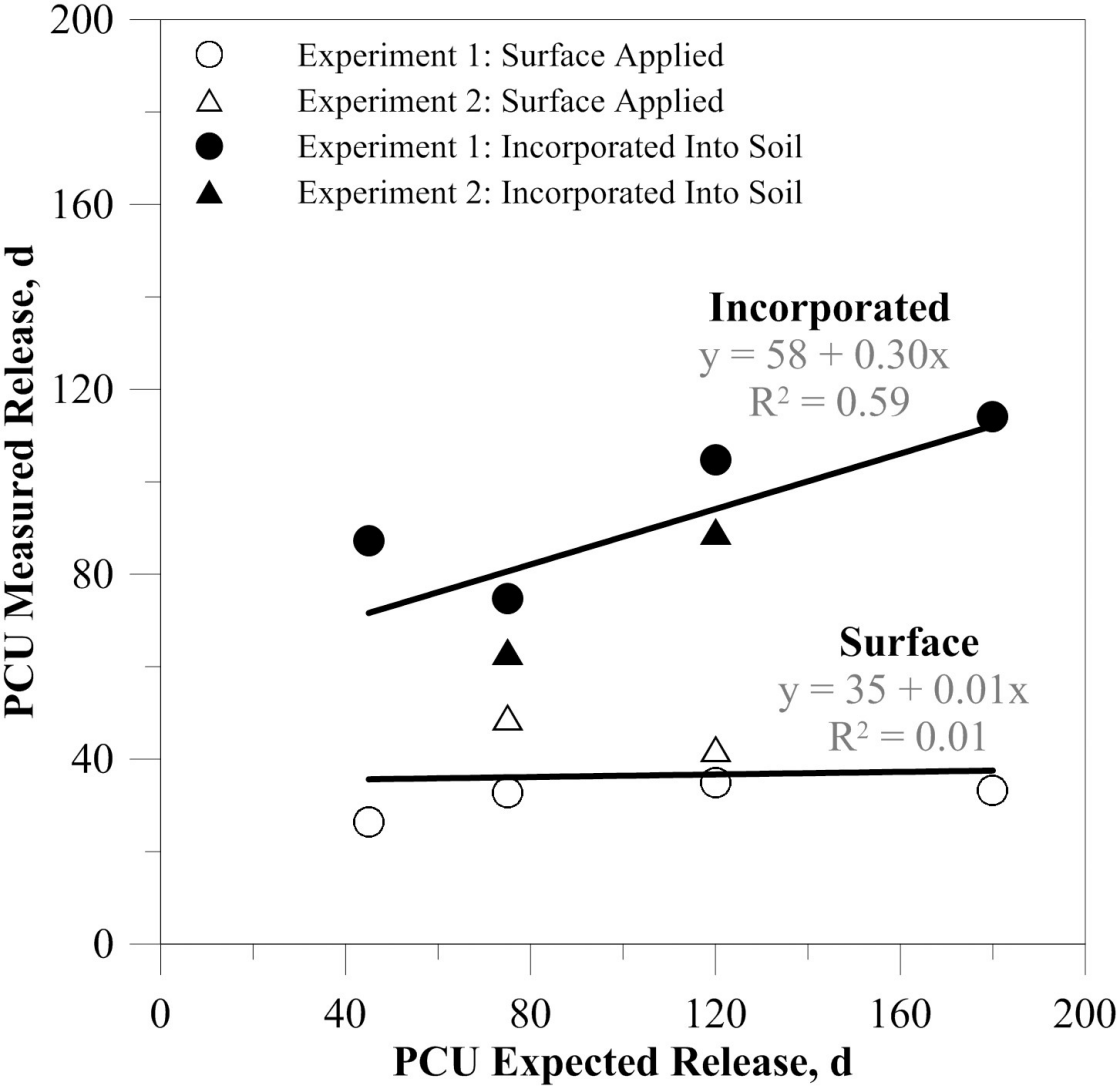
Measured vs. expected N release from (PCU) polymer-coated urea products with varying release timings. Fertilizers were applied to the soil surface or incorporated into the soil under fluctuating daily temperatures. The expected values were those listed by the manufacturer as the number of days to release 80% N at 20°C (e.g., PCU 45 = 45 d). The measured values were the estimated number of days it took to reach 80% N release based on the intercept and slope coefficients of each PCU source and treatment scenario from experiments 1 and 2 (Tables 1 and 2).

The results of this study show that PCU fertilizers can roughly match their expected release timings when incorporated into the soil under field conditions. For example, the PCU 45 and 75 products similarly had extended-release with both releasing > 80% of their N nine days after their stated release timing. However, the gap between expected and observed release seems to widen with longer times. For example, the PCU 120 product had > 80% of its N release by 98 d after application, but the PCU 180 d product released by 112 d—gaps of 22 and 68 d, respectively. Despite the lack of perfection, these extended-release timings are accomplishing their stated purpose in controlling the release of N through time, with a moderate correlation between stated and measured timings (Fig 6).

In contrast, when PCU products are applied to the soil or thatch surface, such as with lawn or pasture grasses or with other perennial crops, the release timings measured in this study do not correlate with the manufacturer stated release timings. All of the PCU products tested released > 80% of their N within 35 d of the application when surface applied under field conditions during the summer heat. In contrast to the buffered internal temperatures when fertilizer is incorporated into the soil, the temperatures experienced on the soil surface vary widely—even more than air temperatures as the surface collects solar radiation. These fluctuations and/or the high daily maximum temperatures cause the granules to release their N prematurely.

Surface temperatures are commonly known to exceed 50°C, which is far above room temperature of the laboratories in which these product release timings are determined by the manufacturer. Thus, it is logical that N would be released more rapidly when surface applied. Regardless of this, the PCU is still found to be very effective in supplying N for an extended period [4,28,29] and in reducing N loss [5,10,19,42]. However, the N release is not enough to last the entire growing season for crops that need late-season N supply, such as lawn and pasture grasses [43].

Many CRF and SRF products claim that a single application can provide a steady supply of N to last the entire season. This can be true sometimes, especially when the fertilizer is incorporated into the soil where it is released at timings close to what is expected [16]. However, because the N release from PCU is strongly related to temperature, application to the soil or thatch surface results in a hastening of N release and a single application is not effective. Buss [43] showed that all the N applied with PCU in a single application, either in spring or fall, was not as good as two or three applications of PCU or when urea was applied monthly (at the same annual N rate). This is in agreement with this data showing that the release of N is too rapid to accommodate a single application for the entire growing season. However, a release of 35 d is enough to spread the N out to meet needs for half of the growing season (under the conditions of this study).

Given that the PCU fertilizers with release timings of just under 35 d are inadequate to be applied just once, it is very unlikely that the SCU and PCSCU products would also function as a single application product. Over two applications would likely be required to meet perennial plant N needs. Further research needs to be performed to evaluate these. And a wider range of products needs to be evaluated to be certain that the ones used herein are not an anomaly.

It is also important to note that temperature not only impacts the N release from the fertilizer, but it also impacts the growth of plants and water relations [44]. Cool-season grasses, such as Kentucky bluegrass, are especially affected by temperature and water relations. Timing for N release needs further study for these grasses to examine N availability needs, N release from PCU, and water relations during the hottest parts of the growing season when daily highs approach 40°C and surface temperatures are far hotter. This study was performed with the N applied in the early summer and the Buss [43] study had the N applied in mid-spring and/or in late summer. Application during the hottest portion of the summer may alter these results due to interactions between temperature, plant growth, and N release from PCU.

### Methodology for measuring N release from coated urea products

Manufacturer estimated release timings rarely match release rates under field conditions. With laboratory bench testing, the granules are placed in a flask that is stirred constantly or periodically during the trial at a constant temperature. This is a convenient method for quickly estimating N release. However, the conditions are far from what is experienced in the field. The temperature, which we have demonstrated is vital to the release, does not fluctuate. This is akin to what the granules are exposed to when incorporated into the soil, but far from what is experienced with a surface application. The solutions in the flasks are not similar chemically to what is in the soil and have minimal or no microbial activity, although it is questionable if PCU is biodegradable or not. We would suggest that the method used herein more closely matches field conditions than laboratory bench tests.

Burying a known amount of fertilizer in a porous bag is another common method used to measure release rates of CRF and SRFs. While bags are cost effective and a better method than laboratory incubations in determining the in-field release of N [21], there are also concerns with this method. One of which is that the bag method concentrates the fertilizer—possibly resulting in negative feedback (slowing the N release) because of equilibrium chemistry or a lack of moisture movement in and out of the bag [22]. The method used in this study, of applying granules directly to the soil, is theoretically more accurate. However, this method was more complicated than taking weights or measuring N release on a laboratory bench. Due to the labor constraints associated with collecting and analyzing individual granules, the method used in this study would be better suited for growth chambers using live turfgrass under different temperature regimes.

In our study, the hanging bag did not release N nearly as quickly as when in soil contact. The rate of release was very low in contrast to other researchers [28,29]. This method is expected to have a lower rate of release as water is often inhibited from moving in and out of the bag. However, the higher rate of release noted in these other studies was a likely result of the humid environments in which the studies were conducted. Whereas the studies reported herein were done under semi-arid conditions with low humidity values. For example, during Experiment 2 the average humidity was 19% with a range of 7–37%. Humidity values were not reported in these other studies cited above, but the typical values observed in these areas are commonly > 80%. This level of humidity may very well allow the granules to absorb enough water to swell, especially inside this closed bag, with subsequent urea release. In contrast, in semi-arid conditions, the minimal amount of moisture in the air limited water diffusion into the granule. This may explain the differences in our findings vs. other researchers.

## Conclusion

To optimize release characteristics of PCU and meet the projected release timings, the fertilizer would need to be incorporated into the soil. However, this is not always possible in cases of perennial crops, no-till, topdressing established crops, and turfgrass which necessitates surface applications. Incorporating PCU fertilizers into the soil resulted in release rates that extended over a longer period compared to surface-applied fertilizers and were reasonably matched the predicted release rates. This is a result of protection from buffered temperature extremes when buried in the soil—resulting in decreased diffusion of urea out of the granule. Fertilizer applied to bare soil surfaces and thatch layers were more exposed to high surface temperatures, which diminished the longevity of all PCU products tested. This is of great importance, as all PCU fertilizer applied to the surface, regardless of the expected release longevity, released ≥ 80% N between 28 and 46 d for both experiments. Although the release on the surface was more rapid than expected, the CRF properties of PCU are still beneficial, but the expectation that release can be extended over an entire growing season is unrealistic for these PCU products under these conditions. The release rates for SCU and PCSCU were concerning for their utilization as SRF and CRF, respectively—although further work is needed to validate these findings. When comparing static temperature conditions of a laboratory and variable temperatures observed in field conditions, fertilizers under static temperature conditions did not perform as expected. Results showed that under these conditions the release of N was slowed. If these parameters are used to estimate release under field conditions, the release would be vastly overestimated. To match release with field conditions, release rates should be estimated under field conditions.

## Supporting information

S1 Data (XLSX)
